# From Cough to Myocarditis: A Systemic Tale of Adult-Onset Still's Disease

**DOI:** 10.7759/cureus.93420

**Published:** 2025-09-28

**Authors:** Iman A. A Shaat, Faiza Javed, Taha Elsahy, Praveenraja Shanmugam, Rabbiya Shafqat Cheema

**Affiliations:** 1 General Medicine, Peterborough City Hospital, Peterborough, GBR; 2 Acute Medicine, Peterborough City Hospital, Peterborough, GBR; 3 General Internal Medicine, Peterborough City Hospital, Peterborough, GBR

**Keywords:** adult-onset still’s disease, hyperferritinemia, myocarditis, polyarthritis, yamaguchi criteria

## Abstract

Adult-onset Still's disease (AOSD) is a rare systemic autoinflammatory disorder that presents a significant diagnostic challenge due to its nonspecific features and overlap with infectious, autoimmune, and malignant conditions. We report a case of a 39-year-old man who initially presented with fever, sore throat, myalgia, and cough. He was initially treated for a presumed respiratory infection. On re-presentation, he developed worsening pyrexia, polyarthritis, odynophagia, and dyspnea. Laboratory investigations revealed markedly elevated inflammatory markers, hyperferritinemia exceeding 6000 µg/L, neutrophilic leukocytosis, anemia, thrombocytopenia, and deranged liver function tests. Imaging demonstrated splenomegaly and pleural effusion, while elevated troponin and ECG changes were consistent with myocarditis. Extensive infectious, autoimmune, and malignant evaluations were unrevealing, and despite broad-spectrum antibiotics, his symptoms persisted. Given the constellation of prolonged fever, systemic inflammation, arthritis, hyperferritinemia, and exclusion of alternative causes, a diagnosis of AOSD was made. The patient responded dramatically to high-dose corticosteroid therapy, with rapid resolution of fever and arthritis and significant biochemical improvement. This case underscores the importance of recognizing AOSD in patients with prolonged pyrexia of unknown origin, particularly when ferritin levels are markedly elevated, and highlights how systemic features such as cardiac and serosal involvement may indicate severe disease. Early initiation of corticosteroids is critical, as timely treatment can lead to rapid recovery and help prevent life-threatening complications such as macrophage activation syndrome.

## Introduction

Adult-onset Still’s disease (AOSD) is a rare, systemic autoinflammatory condition characterized by quotidian fevers, polyarthritis, and an evanescent rash. The disease was first described in 1971 by Bywaters, who documented 14 adult patients aged 17 to 35 with clinical similarities to the childhood form of Still’s disease [1]. The term "Still’s disease" originally referred to a systemic-onset juvenile idiopathic arthritis (SoJIA), and due to overlapping features in gene expression and clinical presentation, some researchers consider SoJIA and AOSD part of a shared disease spectrum [2].

AOSD presents a diagnostic challenge due to its nonspecific symptoms and overlap with infectious, autoimmune, and neoplastic diseases. It is predominantly a diagnosis of exclusion, with no pathognomonic test, though hyperferritinemia and systemic inflammation are hallmark laboratory findings. Delayed diagnosis is common, potentially leading to significant morbidity.

## Case presentation

Medical history and demographics 

A 39-year-old man of Indian origin presented to the emergency department with a seven-day history of pyrexia, generalized myalgia, productive cough, sore throat, and rhinorrhea. Clinical examination on initial review was unremarkable. Laboratory investigations revealed elevated inflammatory markers, while chest radiography was normal. A presumptive diagnosis of an upper respiratory tract infection was made, and he was discharged with a course of oral antibiotics. 

He re-presented five days later with worsening symptoms, particularly arthralgia, odynophagia leading to reduced oral intake, dry cough and increasing breathlessness. He did not report any history of orthopnea, recent travel, contacts with sick individuals, or any significant personal or family medical history. He was fully vaccinated, a lifelong non-smoker, and reported minimal alcohol consumption. He worked in the Information Technology sector and had last traveled to India two years earlier. He had a past medical history of childhood asthma, but was otherwise fit and well.

The clinical examination was positive for a lethargic appearance requiring assistance to mobilize, fever of 38.8°C, tachycardia of 118 bpm, tachypnea of 21 breaths/min and mild bilateral expiratory wheeze at the apices. 

Investigations 

**Figure 1:**
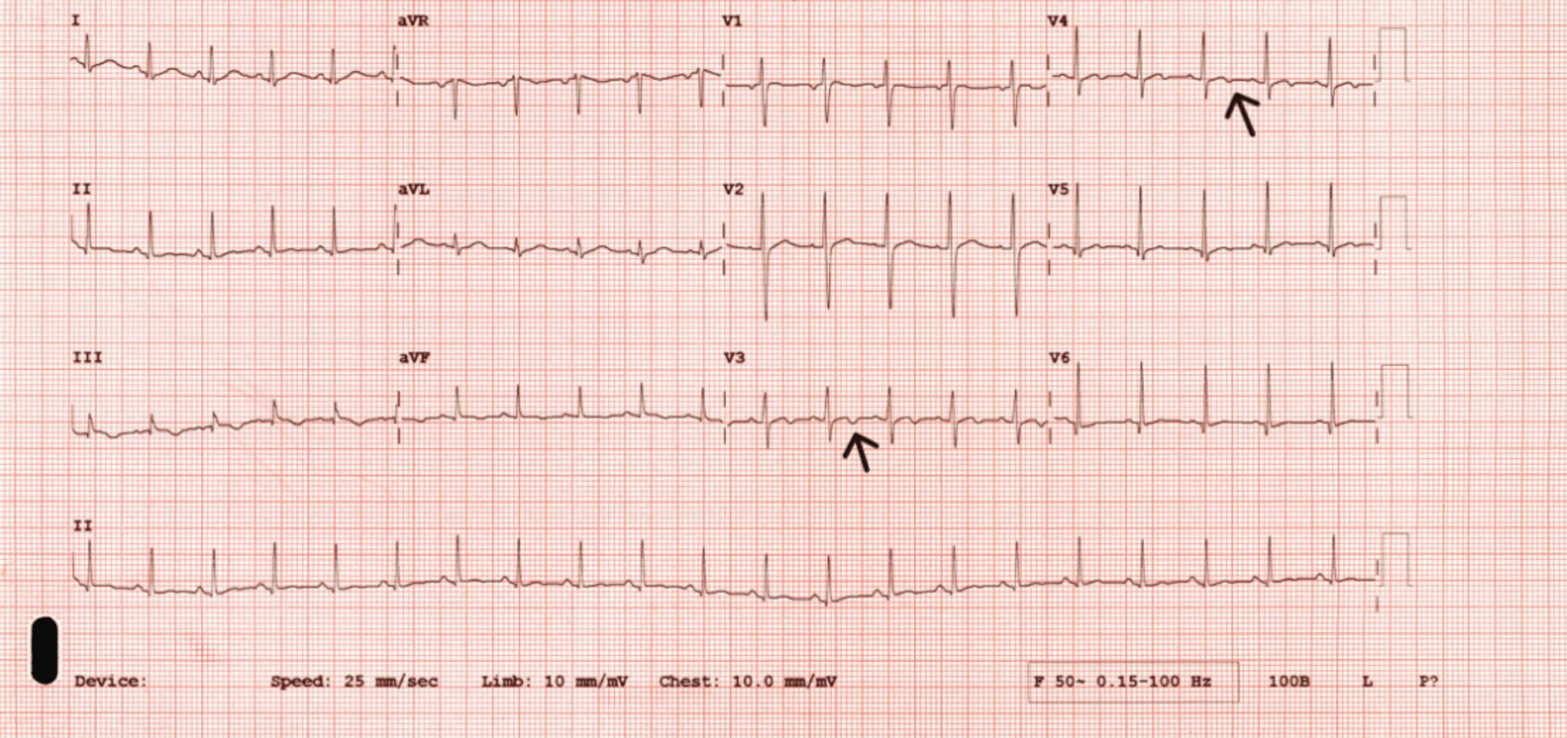
Electrocardiogram (ECG) demonstrating T-wave inversion in precordial leads V3 and V4. Black arrows pointing to the T-wave inversion in precordial leads V3 and V4.

Initial laboratory investigations revealed markedly elevated inflammatory markers, including a C-reactive protein (CRP) level of 276 mg/L, erythrocyte sedimentation rate (ESR) of 127 mm/h, and a serum ferritin of 6111 µg/L. The complete blood count demonstrated neutrophilic leukocytosis (white cell count (WCC) 15.0 × 10⁹/L), anemia (Hb 110 g/L), and borderline thrombocytopenia (platelets 125 × 10⁹/L). Liver function tests were mildly deranged, and hyponatremia was also noted (Table 1). Troponin levels were elevated, and an electrocardiogram (ECG) showed T-wave inversion in leads V3-V4 (Figure 1). Despite the absence of chest pain, a provisional diagnosis of myocarditis was made following cardiology input. An echocardiogram was unremarkable. Serial troponin measurements showed a peak on day six and seven, followed by a marked decline after initiation of corticosteroid therapy, supporting the inflammatory myocardial involvement rather than an ischemic etiology (Table 2). A cardiac MRI was considered to further evaluate for myocardial inflammation and fibrosis; however, this could not be performed as the patient elected to travel back to India permanently.

**Table 1:** Initial blood test results.

Blood parameters	Results	Reference range
White cell count (10^9^/L)	15.0	4-11
Hemoglobin (g/L)	110	130-180
Platelet count (10^9^/L)	125	150-400
C-reactive protein (mg/L)	276	<5
Erythrocyte sedimentation rate (mm/h)	127	<20 (men), <30 (women)
Ferritin (µg/L)	6111	30–400 (men), 15–150 (women)
Total bilirubin (µmol/L)	16	0-21
Alanine aminotransferase (IU/L)	141	<41
Alkaline phosphatase (IU/L)	200	30-130
Cardiac troponin T (ng/L)	183	<12
Sodium (mmol/L)	132	133-146

**Table 2:** Serial troponin measurements during admission and in relation to steroid therapy.

Day	Troponin (ng/l)
1	7
6	183
7	176
9	17
After steroids	7

**Figure 2:**
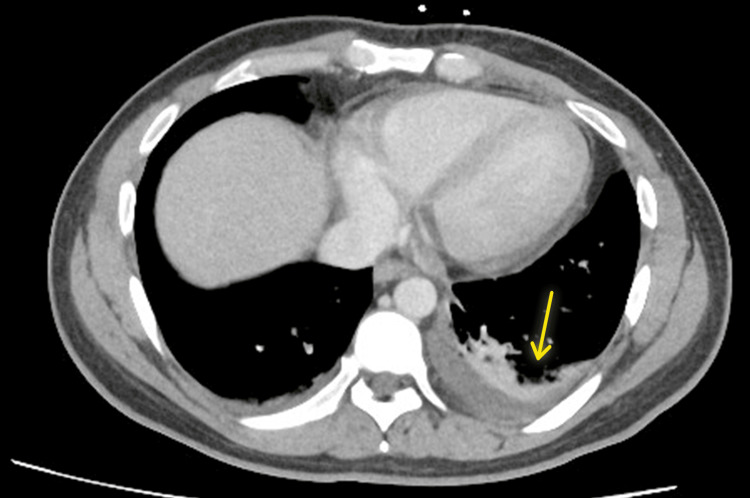
Computed tomography of the chest (axial cut) demonstrating a small left-sided pleural effusion with underlying atelectasis (yellow arrow). Yellow arrow showing the pleural effusion and atelectasis.

Given the presence of tachycardia, dyspnea, and an elevated D-dimer, a CT pulmonary angiogram (CTPA) was performed, which showed no evidence of pulmonary embolism but revealed bilateral basal atelectasis and a small left-sided pleural effusion, which resolved spontaneously before diagnostic aspiration could be performed (Figure 2). 

Empirical treatment with intravenous piperacillin-tazobactam and intravenous fluids was initiated. However, the patient continued to experience fevers that spiked over the following six days. Hematology was consulted due to the markedly elevated ferritin level. Although hemophagocytic lymphohistiocytosis (HLH) was ruled out based on clinical and laboratory criteria, macrophage activation was suspected.

During admission, the patient developed a painful, swollen left knee, which later shifted to the right knee with a moderate effusion. Doppler ultrasound excluded deep vein thrombosis (DVT). X-ray imaging of the joints was unremarkable. Joint aspiration was performed and was negative for crystals and organisms. In the absence of a clear cause, a presumptive diagnosis of reactive arthritis was considered. Antibiotic therapy was escalated to intravenous meropenem, but there was no clinical response. Cultures from blood, urine, sputum, and synovial fluid all remained negative. 

Antinuclear antibody (ANA), extractable nuclear antigen (ENA), rheumatoid factor (RF), and anti-neutrophil cytoplasmic antibodies (ANCA) were all negative. Viral serologies for HIV, hepatitis B and C, cytomegalovirus (CMV), and Epstein-Barr virus (EBV) were negative. Stool cultures, sputum cultures, and atypical pneumonia screens were similarly non-revealing. Tests for brucellosis and visceral leishmaniasis were negative. Tuberculosis was excluded with a negative Quantiferon-TB Gold assay, negative acid-fast bacilli (AFB) cultures, and an unremarkable bronchoalveolar lavage (BAL) following bronchoscopy conducted with infectious disease input. 

**Figure 3:**
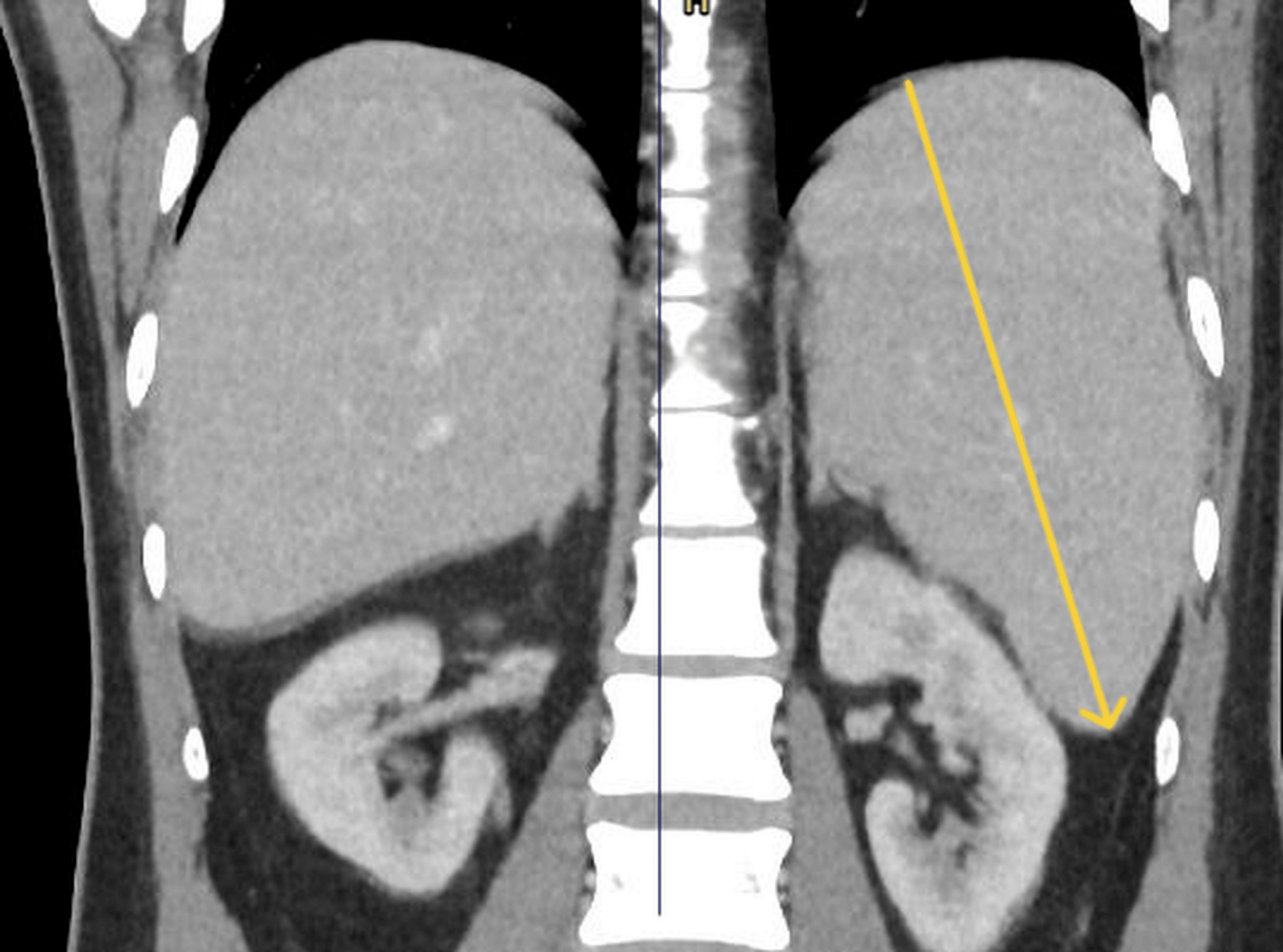
Computed tomography of the abdomen (coronal view) showing splenomegaly. Yellow arrow showing the splenomegaly.

A CT scan of the chest, abdomen, and pelvis (CT CAP) was performed to evaluate for occult malignancy or deep-seated infection. No mass lesions, lymphadenopathy, or abscesses were identified. The only significant finding was splenomegaly measuring 16.5 cm (Figure 3). This, in combination with persistent pyrexia, systemic inflammation, arthritis, and markedly elevated ferritin levels, prompted consideration of systemic autoinflammatory syndromes. 

Treatment 

With infectious, autoimmune, thromboembolic, and malignant causes thoroughly excluded, the diagnostic focus shifted toward adult-onset Still’s disease (AOSD) and macrophage activation syndrome as unifying diagnoses after discussion with the rheumatology team. Antibiotics were discontinued, and the patient was commenced on high-dose prednisolone (60 mg daily). 

Outcome and follow-up 

The patient showed a dramatic clinical improvement within 48 hours of corticosteroid initiation. Fevers resolved, inflammatory markers fell significantly, and joint symptoms improved. He was discharged with a tapering steroid plan. He was reviewed one month later in the rheumatology clinic, where both his symptoms and blood parameters had shown significant improvement. 

## Discussion

As is common in AOSD, the diagnostic process was complicated by a broad differential and overlapping features with more prevalent infectious and autoimmune conditions, resulting in delayed diagnosis and prolonged empirical antibiotic use. 

The incidence of AOSD is low, with reported rates ranging between 0.16 and 0.4 per 100,000 population per year. It exhibits a bimodal age distribution, with peaks of onset typically occurring between 16-25 years and 36-46 years. A five-year retrospective national study in the United States reported an inpatient mortality rate of 2.6% [3]. Interestingly, our patient was also within this predicted age group at presentation. 

This case exemplifies the diagnostic complexity of AOSD. The patient initially presented with a nonspecific febrile illness and upper respiratory tract symptoms, mimicking a self-limiting infection. As his condition evolved, he developed features suggestive of systemic inflammation and polyarthritis, yet extensive investigations failed to reveal an infectious, autoimmune, or neoplastic cause. 

AOSD is primarily a diagnosis of exclusion, and clinical criteria, such as the Yamaguchi and Fautrel systems, aid in diagnosis in the absence of pathognomonic tests [4]. Our patient fulfilled several major Yamaguchi criteria, including quotidian fever for more than one week, arthralgia, leukocytosis, and sore throat, in addition to minor criteria such as abnormal liver function and negative autoantibodies (Table 3).

**Table 3:** Yamaguchi criteria for adult-onset Still's disease.

Category	Criteria	Patient findings (Met/not met/not applicable)
Major criteria	Fever ≥39°C lasting ≥1 week	Met
	Arthralgia/arthritis lasting ≥2 weeks	Met
	Typical nonpruritic, salmon-colored rash	Not applicable (difficult to say in darker skin tones)
	Leukocytosis (≥10,000/µL with met ≥80% granulocytes)	Met
Minor criteria	Sore throat	Met
	Lymphadenopathy and/or splenomegaly	Met
	Abnormal liver function tests	Met
	Negative test results for antinuclear antibody (ANA) and rheumatoid factor (RF)	Met
Exclusion criteria	Infections, malignancies (especially lymphoma), other rheumatic diseases (e.g., polyarteritis nodosa)	Met

Although the presence of a characteristic evanescent salmon-pink rash is one of the hallmark features of AOSD, it was not clearly observed in this patient. This highlights an important diagnostic challenge, as erythematous or subtle rashes can be more difficult to detect in patients with darker skin tones, a limitation that may contribute to under-recognition or underestimation of cutaneous signs in clinical practice [5]. 

The markedly elevated ferritin (persistently >6000 µg/L) was a pivotal clue, as hyperferritinemia is a hallmark of AOSD. While not specific, ferritin levels exceeding 3000-5000 µg/L should prompt consideration of AOSD or macrophage activation syndrome (MAS) [6,7]. 

This case was further complicated by the presence of myocarditis and pleurisy, both recognized but less common manifestations of AOSD. Myocarditis was suspected based on elevated cardiac troponins and ECG abnormalities, despite the absence of chest pain. The small pleural effusions noted on imaging likely reflected underlying pleuritic inflammation. These systemic features further support the diagnosis of AOSD and highlight the importance of recognizing them as potential clinical manifestations [8]. 

Corticosteroid therapy remains the mainstay of treatment in AOSD and is often associated with rapid clinical and biochemical improvement, as seen in the case of our patient. Early identification is critical, as delayed diagnosis may increase the risk of serious complications, including pericarditis, liver dysfunction, disseminated intravascular coagulation, and MAS [8,9]. For steroid-refractory or relapsing disease, disease-modifying antirheumatic drugs (DMARDs) or biologics targeting IL-1 or IL-6 pathways may be required [10]. 

## Conclusions

This case illustrates the diagnostic complexity of adult-onset Still’s disease (AOSD), where overlapping features with infection, autoimmune, and malignant conditions delayed recognition. Marked hyperferritinemia with myocarditis, pleurisy, and splenomegaly supported the diagnosis, while subtle rash in darker skin tones added to the challenge. The patient responded rapidly to corticosteroids, underscoring the importance of early recognition and treatment. Clinicians should consider AOSD in patients with pyrexia of unknown origin and systemic inflammation, particularly when ferritin is markedly elevated.

## References

[1] Bywaters EG (1971). Still's disease in the adult. Ann Rheum Dis.

[2] Tomaras S, Goetzke CC, Kallinich T, Feist E (2021). Adult-onset Still's disease: clinical aspects and therapeutic approach. J Clin Med.

[3] Giacomelli R, Ruscitti P, Shoenfeld Y (2018). A comprehensive review on adult-onset Still's disease. J Autoimmun.

[4] Efthimiou P, Kontzias A, Hur P, Rodha K, Ramakrishna GS, Nakasato P (2021). Adult-onset Still's disease in focus: clinical manifestations, diagnosis, treatment, and unmet needs in the era of targeted therapies. Semin Arthritis Rheum.

[5] Lester JC, Taylor SC, Chren MM (2019). Under-representation of skin of colour in dermatology images: not just an educational issue. Br J Dermatol.

[6] Meijvis SC, Endeman H, Geers AB, Ter Borg EJ (2007). Extremely high ferritin levels as diagnostic tool in adult-onset Still's disease. Neth J Med.

[7] Rosário C, Zandman-Goddard G, Meyron-Holtz EG, D'Cruz DP, Shoenfeld Y (2013). The hyperferritinemic syndrome: macrophage activation syndrome, Still's disease, septic shock and catastrophic antiphospholipid syndrome. BMC Med.

[8] Gerfaud-Valentin M, Jamilloux Y, Iwaz J, Sève P (2014). Adult-onset Still's disease. Autoimmun Rev.

[9] Efthimiou P, Kontzias A, Ward CM, Ogden NS (2007). Adult-onset Still's disease: can recent advances in our understanding of its pathogenesis lead to targeted therapy?. Nat Clin Pract Rheumatol.

[10] Ruscitti P, Cipriani P, Ciccia F (2017). Prognostic factors of macrophage activation syndrome in adult patients with adult-onset Still’s disease. Clin Exp Rheumatol.

